# Agreement between equations-estimated resting metabolic rate and indirect calorimetry-estimated resting metabolic rate in low-income obese women

**DOI:** 10.20945/2359-3997000000226

**Published:** 2020-03-30

**Authors:** Isabele Rejane de Oliveira Maranhão Pureza, Mateus Lima Macena, André Eduardo Silva, Dafiny Rordrigues Silva Praxedes, Lais Gomes Lessa Vasconcelos, Telma Maria Menezes Toledo Florêncio, Nassib Bezerra Bueno

**Affiliations:** 1 Faculdade de Nutrição Universidade Federal de Alagoas Maceió AL Brasil Faculdade de Nutrição, Universidade Federal de Alagoas, Maceió, AL, Brasil

**Keywords:** Energy metabolism, basal metabolism, indirect calorimetry, obesity

## Abstract

**Objectives:**

Indirect calorimetry is established as a gold standard to determine the resting metabolic rate (RMR), however, its clinical use is limited, especially in low-income settings. Thus, the use of predictive equations appear as an alternative to estimate the RMR, but its precision is debatable, especially in obese individuals and in populations without specifically developed equations. To evaluate the agreement between the RMR estimated by equations and by indirect calorimetry in low-income obese women.

**Subjects and methods:**

A cross-sectional study with adult and obese women, which estimated the RMR by indirect calorimetry and compared with 13 predictive equations using the concordance correlation coefficient, root mean square error (RMSE) and Bland-Altman methods. The maximum allowed differences were predefined as 10%.

**Results:**

No equation presented its confidence intervals for the Bland-Altman limits of agreement inside the predefined acceptable range. The Harris-Benedict equation achieved better agreement (bias of 2.9% and RMSE of 274.3kcal) whereas the Henry-Rees equation achieved better precision (42.3% of the sample within the 10% maximum allowed difference).

**Conclusion:**

None of the studied equations satisfactorily estimated the RMR estimated by indirect calorimetry. In the absence of specific equations for this population, the use of the Harris-Benedict and Henry-Rees equations could be considered.

## INTRODUCTION

Obesity is a worldwide public health problem and is associated with increased morbidity and mortality (1). In Brazil, data from a national survey revealed that the prevalence of obesity in women with up to 8 years of schooling is 27.8%, while in those with 12 or more years of schooling it is 14.4%, highlighting that women in the lowest stratum of schooling, and presumably income, are the most vulnerable group for the development of obesity (2). When compared to countries from the Organization for Economic Cooperation and Development, the obesity prevalence of 25.5% in low-income Brazilian women would be ranked the 8th highest (3).

Despite the complexity of the various physiological mechanisms and social phenomena that influence the establishment of obesity, most of them culminate in greater individual energy intake in relation to energy expenditure (EE) (4). Hence, for the prevention or treatment of obesity, it is important to adequately determine both energy intake and EE. EE involves basal metabolic rate, thermic effect of food or diet-induced thermogenesis, and physical activity, which may be influenced by several factors, including age, body composition, body and ambient temperature, health condition, use of medications, thyroid hormones, and catecholamines (5). In addition, the environment in which the individual is inserted may influence his or her EE, as some studies have reported an inverse association between socioeconomic status and sympathetic nervous system activity by an increase in the circulating catecholamine and cortisol levels (6). Furthermore, it was recently reported that lower socioeconomic status, alongside higher psychosocial stress and systemic inflammation, induces a greater activity of the cerebellar tonsils, which is considered a measure of stress associated with neural activity (7). Another factor that may influence the resting metabolic rate (RMR) in this population is a possible perinatal malnutrition, which can lead to important metabolic adaptations in adulthood, manifested as short stature, which is common in many developing countries that have experienced nutritional transition and is associated with obesity, especially in low-income populations (8). Early malnutrition is believed to influence energy homeostasis, leading to a reduction in energy requirements and central nervous system modifications that may facilitate fat accumulation (9).

An adequate evaluation of the individual EE is usually achieved by estimating the RMR, which may be calculated through the use of predictive equations or determined by indirect calorimetry (IC), which must be combined with the physical activity level to determine the total EE of the individual (5). Although IC is established as the gold standard for the determination of RMR, its clinical use is limited, especially in low-income settings, because it is a costly method, in addition to limitations related to the qualification of the personnel and logistic issues (10), making its use almost impossible in socially vulnerable populations. In this way, the use of predictive equations appears to be a feasible alternative to estimate RMR, considering that it commonly demands trivial individual parameters such as age, weight, and height. However, the choice of an accurate predictive equation, especially for obese individuals, is still debatable because existing equations prove inadequate to precisely predict RMR because their results become less accurate as body mass index (BMI) increases (11). In addition, the heterogeneity between the studied populations used to derive the equations and the populations for which predictive equations need to be used most, such as low-income ones, may further increase the imprecision of these equations.

Therefore, the present study aimed to determine the predictive equation of RMR that shows the highest agreement with RMR obtained by IC in socially vulnerable obese Brazilian women.

## SUBJECTS AND METHODS

The research was approved by the Research Ethics Committee of the Federal University of Alagoas (number 2 535.99). All participants were informed about the procedures and signed a written informed consent form before starting the study, marking their formal participation. The present study is a substudy of a randomized clinical trial still in progress, registered in the Registro Brasileiro de Ensaios Clínicos (ReBEC) under number RBR-387v6v.

### Population and sample

Obese women, aged 19-44 years and classified as economic class “C” and “D-E”, as determined by the Critério de Classificação Econômica Brasil (CCEB), Brazil’s economic classification criteria, were included (12). The CCEB is an instrument consisting of questions about assets, household employees, housing data, head of household instruction, and access to piped water and paved streets, where each item yields a different score. According to the achieved score, individuals are classified into one of 6 classes that vary from “A”, the highest, to “D-E”, the lowest. Also, data on race were collected, and participants self-reported whether they considered their skin color to be white (Caucasian), black (African descent), brown, yellow (Asian), or indigenous. Obesity was defined by the presence of two of the following criteria: BMI ≥ 30 kg/m^2^ and < 45 kg/m^2^, waist circumference (WC) ≥ 88 cm, body fat percentage ≥ 35%. Women who were on chronic medications (antidiabetic, antihypertensive, antiretroviral, immunosuppressive, antidepressant), experiencing menopause, pregnant or breastfeeding, or had undergone any surgical intervention for weight loss were not included. Sampling was non-probabilistic for convenience, and recruitment occurred through advertisements in the community or direct invitation to women who had some link with the Center for Recovery and Nutritional Education (CREN-AL), which treats malnourished children, located in the 7th administrative region of the municipality of Maceió-AL and has the lowest Human Development Index (HDI) of the municipality (0.65).

### Anthropometric evaluation and body composition

An anthropometric evaluation was performed with weight and height data collection. The participants’ weight was measured on a digital scale and their height was measured by means of a standardized wall stadiometer. BMI was calculated and classified according to the World Health Organization. The percentage of body fat was estimated by means of four-pole electric bioimpedance Sanny BI 1010 (Sanny, São Paulo, SP). For the test, 4 electrodes were fixed in the right hemibody of the patients, who were lying in the supine position, wearing light clothes, barefoot, and without metallic props (13). Participants were instructed not to perform any physical activity, to abstain from drinking in the 24 hours prior to the test, and to undergo a 10-hour fast.

### Estimation of the RMR by IC

RMR was estimated using a gas analyzer (Quark, Cosmed, Rome, Italy). The participants were taken by car to the Federal University of Alagoas Laboratory of Applied Sports Sciences. The collection took place in the morning (between 07:00 and 09:00), in a quiet environment, with low light and a comfortable temperature for the participants (22-26°C), following the same preparation used for bioimpedance, because the measurements were performed at the same moment. The equipment was calibrated before each test session according to the manufacturer’s specifications, with gases in the concentration of 20.9% O_2_ and 5% CO_2_, and a 3 L syringe, with the secondary pressure gauge adjustable between 40 and 60 psi.

On this occasion, measurements of axillary temperature using a digital thermometer and heart rate using a tensiometer were collected to avoid calorimeter measurements in individuals with signs of hyperthermia (> 37.5ºC) or tachycardia (>100 bpm). Participants were asked to wear the equipment’s silicone mask, and thus, the inspired volumes of expired oxygen and carbon dioxide were counted for 15 minutes. Measurements for the first five minutes were discarded to avoid discrepancies due to the location and use of the silicone mask, and data were collected every minute (14). After measuring the oxygen and carbon dioxide volumes in liters per minute, the equation proposed by Weir was used to estimate the RMR.

## ESTIMATION OF THE RMR BY PREDICTIVE EQUATIONS

The studied equations were selected based on the clinical practice use for obese women and those specifically developed for the Brazilian public. Thirteen equations were included: those proposed by Anjos and cols. (15), Bernstein and cols. (16), FAO/WHO/UNU (17), Harris-Benedict (18), Henry-Rees (19), Horie and cols. (20), Mifflin and cols. (21), Owen and cols. (22), Oxford (23), Rodrigues and cols. (24), Schofield (25), Siervo and cols. (26), and Weijs and Vansant (27). In the present study, we used equations that estimate the basal metabolic rate (BMR) or the RMR because these are used for the same purpose in clinical practice and often used interchangeably in scientific studies (5). Information on equation formulas is given in Table 1.

**Table 1:** Predictive equations used to estimate the Resting Metabolic Rate in obese women found in the literature (n = 13)

Equation	Year	Formula
Anjos et al. (15)	2014	(37.46 x Weight (kg)) + (37.13 x Height (cm)) – (2.92 x Age (years)) – 3407.09
Bernstein et al. (16)	1983	(7.48 × Weight (kg)) – (0.42 × Height (cm)) – (3 × Age (years)) + 844
FAO/WHO/UNU (17)	2001	a.18 – 30 years: (14.818 × Weight (kg)) + 486.6 b.31 – 60 years: (8.16 × Weight (kg)) + 845.6
Harris-Benedict (18)	1919	655.0955 + (9.5634 × Weight) + (1.8496 × Height (cm)) – (4.6756 × Age (years))
Henrry-Rees (19)	1991	a.18 – 30 years: (0.048 × Weight (kg)) + (2.562 × 239) b.31 – 60 years: (0.048 × Weight (kg)) + (2.448 × 239)
Horie et al. (20)	2011	560.43 + (5.39 × Weight (kg)) + (14.14 × Free Fat Mass (kg))
Mifflin et al. (21)	1990	(9.99 × Weight (kg)) + (6.25 × Height (cm)) – (4.92 × Age (years)) – 161
Owen et al. (22)	1986	795 + (7.18 × Weight (kg))
Oxford (23)	2005	a. 18 – 30 years: (10.4 × Weight (kg)) + (615 × Height (m)) – 282 b. 31 – 60 years: (8.18 × Weight (kg)) + (502 × Height (m)) – 11.6
Rodrigues et al. (24)	2010	a. IMC<35 kg/m^2^: 407.57 + (9.58 × Weight ) + (2.05 × Height (cm)) – (1.74 × Age (years)) b. IMC>35 kg/m^2^: 172.19 + (10.93 × Weight) + (3.10 × Height (cm)) – (2.55 × Age (years))
Schofield (25)	1985	a. 18 – 30 years: (0.062 × Weight (kg) + 2.036) × 239 b. 31 – 60 years: (0.034 × Weight (kg) + 3.538) × 239
Siervo et al. (26)	2003	(11.5 × Weight (kg)) + 542.2
Weijs & Vansant (27)	2010	(Weight (kg) × 14.038) + (Height (cm) × 4.498) – (Age (years) × 0.977) − 221.631

### Physical activity

The physical activity level was measured using a triaxial accelerometer (activPAL^®^, Glasgow, UK), which was placed in the frontal area of the participants’ thigh, in the medium point between the inguinal line and the upper edge of the patella, with two transparent, hypoallergenic medical dressings (VitaMedical^®^, Minas Gerais Brazil) to avoid contact of the device with the skin of the participants. The women used the accelerometers for 3 consecutive days without removal for any activity. The data were transferred to the activPAL3™ software version 7.2.32 to yield the intensity and duration of each activity performed by the individuals. The system estimates the physical activity for the period in which the device is used, and the calculation is based on acceleration from three body axes – anteroposterior, lateral, and vertical – by means of computing the periods in which the individual was lying down/sitting down, standing, walking, and running at every tenth of a second. The activPAL software provides an indirect estimate of the Metabolic Equivalent of Tasks (METs) based on default values for sitting/lying (1.25 MET), standing (1.40 MET), and stepping at 120 steps per minute (4 MET). For cadences that differ from 120 steps per minute, the following equation is used to calculate the MET estimate: MET.h = (1.4 xd) + (4 - 1.4) x (c / 120) x d, where c is the cadence (steps per minute) and d is the duration of the activity (in hours). Software analysis of accelerometer data provides the MET value for the entire period in which individuals used the device, multiplying the MET value for each activity by the duration of the activity. MET is defined as the amount of oxygen consumed while at rest, which corresponds to 3.5 ml of O_2_ per kg of body weight x min, or as 1 kcal/kg/hour, and is roughly equal to the cost of sitting quietly. This concept, while simple, can express the EE of physical activity as a multiple of RMR, regardless of the individual’s characteristics and type of activity.

### Statistical analysis

The methods for assessing the agreement between the equation-RMR and the IC-RMR were as follows: (a) First, the method proposed by Bland and Altman (28), where the percentage differences were used to reduce the proportionality bias. Concordance limits and their 95% confidence intervals were calculated, and the maximum allowed difference was predefined as an acceptable limit of agreement of ± 10% (29,30), which was also used to determine the precision (i.e., the percentage of participants with the equation-RMR result with a bias lower than 10% compared to the IC-RMR). In addition, to evaluate which equation-RMR presented no significant bias in relation to the IC-RMR, a t-test for paired samples was performed. (b) Second, the correlation concordance coefficient (CCC), obtained by multiplying the Pearson’s correlation coefficient by the accuracy (deviation between the 45º line and the best fit line) for each pair, was calculated. The CCC is generally classified as poor (≤ 0.20), fair (0.21-0.40), moderate (0.41-0.60), good (0.61-0.80), or very good (0.81-1.0). (c) Third, the root mean square error (RMSE) between the IC-RMR and each equation-RMR was determined, with the interpretation that lower values show better agreement between the methods. In addition, to explore the influence of race, BMI, and MET.hour on the bias of each equation, we conducted a Kruskal-Wallis test, Pearson correlations, and Spearman correlations. To observe the influence on weight-adjusted RMR, multivariable linear regression was performed. All analyses were performed using the statistical package MedCalc Statistical Software v. 16.4 (MedCalc Software bvba, Oostende, Belgium), and an alpha value of 5% was adopted.

With regard to sample size, because this study used the baseline data of a randomized clinical trial, the sample size calculation was not delineated considering the present analyses. An *a posteriori* calculation, based on the equation with the lowest RMSE in the present study, and that considered the mean and the standard deviation of the differences between the estimated-RMR and the IC-RMR, a power of 80%, an alpha of 5%, and the present sample size of 59 was conducted to estimate the maximum allowed difference of the limit of agreement that should be considered in the present study.

## RESULTS AND DISCUSSION

**Figure 1:**
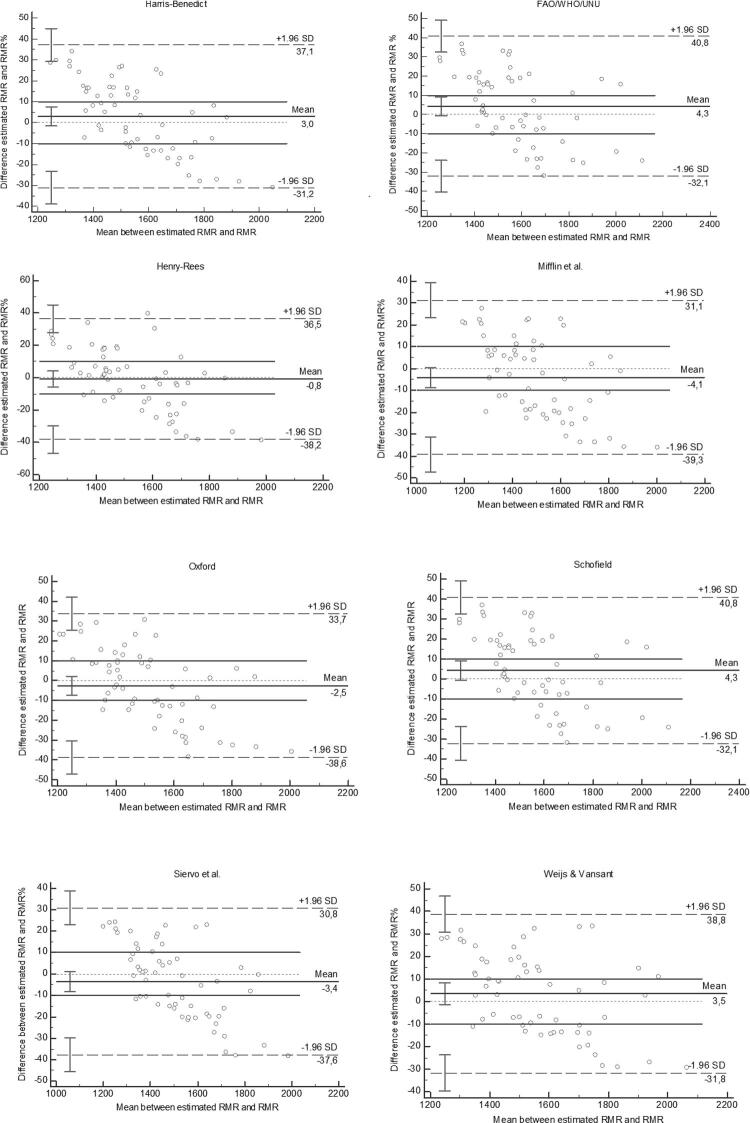
Bland-Altman plots of differences in resting metabolic rate (RMR), measured using indirect calorimetry and calculated using predictive equations that presented no significant bias.

Fifty-nine obese women were included, and their characteristics are presented in Table 2. The assessment of the agreement between the RMR by the predictive equations and the RMR measured by the IC of the women is presented in Table 3. Among the 13 equations analyzed, a significant bias was observed in five: those proposed by Anjos and cols. (15), Bernstein and cols. (16), Horie and cols. (20), Owen and cols. (22), and Rodrigues and cols. (24). In addition, no equation presented limits of agreement within the predefined acceptable range of ± 10%. The equations that showed nonsignificant bias were those proposed by FAO/WHO/UNU (17), Harris-Benedict (18), Henry-Rees (19), Mifflin and cols. (21), Oxford (23), Schofield (25), Siervo and cols. (26), and Weijs and Vansant (27). The equation proposed by Henry-Rees (19) presented the lowest bias (0.8%) and the highest precision (42.3%) but also the lowest CCC. The other equations presented a reasonable CCC, that of Weijs and Vansant (27) being the highest (0.27). The Harris-Benedict equation (18) presented the lowest RMSE values. Using the data obtained with this equation in the *a posteriori* calculations of the maximum allowed difference, a value of 750 kcal was found, which is roughly 50% of the IC-RMR of the sample, whereas a maximum allowed difference of 10% was predefined. The Bland-Altman scatter plots can be found in Figure 1.

**Table 2:** Characteristics of included women (n = 59)

Variables	Mean	Standard deviation
Age (years)	31.57	7.01
Weight (kg)	80.86	11.60
Height (m)	1.55	0.06
BMI (kg/m^2^)	32.86	5.94
Free Fat Mass (kg)	45.02	4.77
Body fat (%)	42.78	5.45
Resting Metabolic Rate (kcal)	1543.93	290.92
MET.hour (24h-multiple of RMR)	1.45	0.06

	**n**	**%**

**Race **		
White	10	16.9
Black	14	23.7
Brown	35	59.3

BMI: body mass index.

**Table 3:** Evaluation of the concordance between resting metabolic rates by equations and resting metabolic rates measured by indirect calorimetry in obese women with social vulnerability (n = 59)

Equation	RMR (kcal)^a^		RMSE^b^	Bias^c^	T-test	Limits of Agreement [LL – UL]^e^	LoA Lower Limit	LoA Upper Limit	CCC^f^	MPE^g^	MNE^h^	Precision^i^

	Mean	SD	kcal	(%)	P^d^	(%)	[CI 95%]	[CI 95%]		(%)	(%)	(%)
RMR-CI	1543.9	290.9	-	-	-	-	-	-	-	-	-	
Anjos et al. (15)	1268.9	138.8	394.7	-18.2	<0.01	[-54.0 – 17.4]	[-62.1– -45.8]	[9.3 – 25.6]	0.12	12.2	-41.0	32.2
Bernstein et al. (16)	1288.8	90.1	372.0	-16.4	<0.01	[-49.9 – 17.1]	[-57.6 – -43.3]	[9.4 – 24.7]	0.11	15.2	-40.5	33.8
FAO/WHO/UNU (17)	1593.97	167.5	296.7	4.3	0.07	[-32.1 – 40.8]	[-40.4 – -23.7]	[32.4 – 49.1]	0.22	45.1	-27.5	33.8
Harris-Benedict (18)	1568.4	125.4	274.7	2.9	0.19	[-31.1 – 37.0]	[-38.9 – -23.3]	[29.2 – 44.8]	0.23	40.9	-26.7	35.5
Henrry-Rees (19)	1511.3	137.5	303.2	-0.8	0.74	[-38.1 – 36.5]	[ -46.7 – -29.6]	[28.0 – 45.0]	0.10	49.3	-32.2	42.3
Horie et al. (20)	1656.6	119.3	292.4	8.4	<0.01	[-25.1 – 42.0]	[-32.8 – -17.4]	[34.3 – 49.7]	0.22	48.3	-24.0	35.5
Mifflin et al. (21)	1463.4	148.3	292.3	-4.1	0.08	[-9.3 – 31.1]	[-47.3 – 31.2]	[23.0 – 39.1]	0.23	31.9	-30.6	33.8
Owen et al. (22)	1375.6	83.3	303.2	-9.9	<0.01	[-43.7 – 23.8]	[-51.4 – -36.0]	[16.1 – 31.6]	0.13	21.7	-38.3	37.2
Oxford (23)	1486.2	134.2	294.3	-2.5	0.30	[-38.6 – 33.7]	[-46.9 – -30.3]	[25.4 – 41.9]	0.16	36.4	-32.1	38.9
Rodrigues et al. (24)	1409	94.4	326.0	7.5	<0.01	[-44.7 – 29.6]	[-53.2 – -36.2]	[21.1 – 38.1]	0.03	41.5	-37.4	35.5
Schofield (25)	1593.9	169.0	296.7	4.3	0.07	[-31.1 – 40.8]	[-40.4 – -23.7]	[32.4 – 49.1]	0.22	45.1	-27.5	33.3
Siervo et al. (26)	1472.1	133.4	284.0	-3.4	0.13	[-37.6 – 30.7]	[-45.4 – -29.7]	[22.9 – 38.5]	0.23	27.6	-32.1	35.5
Weijs & Vansant (27)	1582.2	178.5	289.4	3.5	0.13	[-31.7 – 38.8]	[-39.8 – -23.6]	[30.5 – 46.8]	0.27	40.2	-25.4	28.8

^a ^Mean estimated RMR.

^b ^Root mean square error.

^c ^Bland-Altman percentage mean differences. Calculated by dividing the difference between the estimated-RMR and RMR-CI by the mean between the estimated RMR and RMR-CI. multiplied by 100.

^d ^P-value for a “t” test for paired samples, comparing the mean estimated-RMR to the mean RMR-CI.

^e ^Lower limit and upper limit of the Bland-Altman Limits of Agreement, where 95% of the differences is expected to lie between.

^f ^Concordance Correlation Coefficient.

^g ^Maximum Positive Error.

^h ^Maximum Negative Error.

^I ^Percentage of participants with predicted resting metabolic rate within 10% of IC measured values.

The present study demonstrated that among the 13 equations analyzed to estimate RMR, seven showed no significant bias when compared to IC-RMR. The Henry-Rees (19) equation showed the lowest bias, and the Harris-Benedict (18) equation showed the highest agreement when evaluated according to the RMSE. However, none of the equations showed limits of agreement narrower than the predefined acceptable range of 10%, indicating that no equation satisfactorily estimated the IC-RMR in the present sample. It is worth mentioning that all the equations developed for the Brazilian population analyzed in this study (Anjos and cols. (15), Horie and cols. (20) and Rodrigues and cols. (24)) presented significant bias, which indicates poor agreement with the IC-RMR.

The Henry-Rees (19) equation was not analyzed in any of the RMR concordance assessment studies on obese women to our knowledge, which prevents the comparability of our finding that this equation present the lowest bias among all equations (11,29,30). A study with Brazilian obese women (29) also showed that the Harris-Benedict equation (18) and the Mifflin (21) equation showed nonsignificant bias compared to the IC-RMR, the Harris-Benedict equation (18) being the most accurate (40%) among the analyzed equations. In a systematic review, which analyzed the most accurate predictive equations of rest and total EE in overweight adults, the equation of Mifflin and cols. (21) showed the lowest bias in the BMI subgroup of 30-39.9 kg/m^2^ (-0.5%), while the Harris-Benedict equation (18) provided a more precise prediction (62.7% predicted at 10% of the measure) for the subgroup with BMI ≥ 30 kg/m^2^ (31). Horie and cols. (20), when comparing it with the IC-RMR, also observed good precision and accuracy of the Harris-Benedict equation (18), while developing a new equation to estimate RMR in severe obesity. In a study carried out in northern Spain with 86 obese individuals, it was observed that the Harris-Benedict equation (18) presented one of the lowest RMSEs (152 kcal/d) among the analyzed equations (31), similar to the present study, in which this equation showed the lowest RMSE.

This evidence suggests that the Harris-Benedict equation (18) is acceptable for individuals with a wide weight range and in several studies with obese individuals (30). This is one of the most used equations in clinical practice, and, because it is the oldest one, it has already undergone extensive validation (16), although some studies support the use of the Mifflin (21) equation for extremely obese men and women, especially in the American population (30,31). Although the Harris-Benedict equation (18) was not developed for obese individuals, it has been reported in other studies that the equations developed for eutrophic individuals are more precise when applied to obese individuals, compared to those equations developed specifically for obese individuals (5,29). In the present study, equations developed specifically for obese individuals, such as those by Weijs and Vansant (27) and Horie and cols. (20), did not perform better than the other equations, despite the former presenting the highest CCC in the present study. Furthermore, equations that included body composition data, such as free fat mass and body fat, did not show greater precision when compared to equations without the use of these variables, as one would expect. This is perhaps due to the possible inaccuracy in obtaining these variables of body composition in the obese population, especially with the use of bioimpedance (29). Hence, the equations based on simple anthropometric parameters, such as weight and height, are more feasible in the outpatient routine, especially in a context of social vulnerability, when compared to the equations based on body composition, because it does not generate additional costs for its application.

The environmental conditions may influence the biological factors of women living in socially vulnerable contexts, a fact corroborated by studies with women from the same community as the present research group (32), which evaluated the association between height and total EE, concluding that women with short stature, possibly due to perinatal malnutrition, presenting the same energy consumption and a higher level of physical activity, showed a lower total EE when compared to women with higher stature. It is noteworthy that the mean height of the women included in the present study was 1.55 m, which is below the expected median height for adult women. In the present study, there was no association between self-reported race, BMI, MET.hour, and the bias presented by the studied equations, as shown in Table 4. There was also no interaction between weight-adjusted RMR and race (p = 0.47), BMI (p = 0.52), and MET.hour (p = 0.13). Sharp and cols. (33) assessed whether there were ethnic and gender differences in RMR in a group of young American adults. The authors concluded that there are differences in RMR between African Americans and white women, but these differences are unlikely to be the main reason for the high rate of obesity in African American women. It is possible that the self-report method adopted in the present study to define race may have induced some bias in the analysis; however, it is the method recommended by the Brazilian Institute of Geography and Statistics. Also, the heterogeneous genetic profile of the Brazilian population has an important contribution from European, African, and Amerindian ancestry, and this process of miscegenation makes it difficult to observe ethnic/racial patterns (34). Regarding BMI, we recognize that the sample was composed by obese individuals including participants with a wide range of BMI, covering the 3 groups defined by the WHO to classify the degree of obesity. However, in our study, BMI did not influence either bias or weight-adjusted RMR, a finding corroborated by a study conducted in Brazil with a sample composed of obese but hospitalized individuals, which aimed to define the best RMR value in kcal/kg, considering the class and/or the BMI range of the patients and observed that there was no difference in RMR values between the different BMI classes in the fasting state (35).

**Table 4:** Interaction analyzes between the bias of each equation, in %, and MET.hour, race and BMI

	MET.hour	Race	BMI
Equation (bias in %)	p-value*	p-value^‡^	p-value^†^
Anjos et al. (15)	0.26	0.63	0.95
Bernstein et al. (16)	0.37	0.39	0.65
FAO/WHO/UNU (17)	0.27	0.55	0.20
Harris-Benedict (18)	0.33	0.42	0.70
Henrry-Rees (19)	0.21	0.49	0.06
Horie et al. (20)	0.47	0.39	0.37
Mifflin et al. (21)	0.12	0.68	0.78
Owen et al. (22)	0.38	0.39	0.53
Oxford (23)	0.22	0.62	0.07
Rodrigues et al. (24)	0.94	0.23	0.09
Schofield (25)	0.27	0.55	0.30
Siervo et al. (26)	0.30	0.45	0.66
Weijs & Vansant (27)	0.18	0.58	0.45

BMI: body mass index.

* p-value for the Spearman correlations. ^‡^ p-value for the Kruskal Wallis test. ^†^ p-value for the Pearson correlations.

A major limitation of the present study is due to the absence of an *a priori* sample size calculation. Considering the sample size of this study, the maximum allowed difference that should have been considered was 750 kcal, or roughly 50% of the IC-RMR, while we assumed a predefined maximum allowed difference of 10%. This means that with the present sample, an estimated RMR that showed limits of agreement within a range of 50% of the IC-RMR would still be considered to agree with the IC-RMR, indicating the low precision yielded by the present sample size. However, because our goal was to show which equation would perform better, considering that there is no gold-standard equation for this population, we believe that the present study may still provide useful information for clinicians and researchers working with populations similar to ours.

In conclusion, none of the studied equations satisfactorily estimated the IC-RMR, which indicates that these equations are not sufficiently precise in the context of this study. However, the Harris-Benedict (18) equation presented the highest agreement, and the Henry-Rees (19) equation presented the highest precision and lowest bias. Therefore, in the absence of specific equations for this population, the use of the Harris-Benedict (18) and Henry-Rees (19) equations could be considered.
